# Downregulation of Vascular Endothelial Growth Factor Enhances Chemosensitivity by Induction of Apoptosis in Hepatocellular Carcinoma Cells

**DOI:** 10.22074/cellj.2016.3730

**Published:** 2015-07-11

**Authors:** Doan Chinh Chung, Le Thanh Long, Hoang Nghia Son, Le Tri Bao, Do Minh Si, Le Van Dong

**Affiliations:** 1 Faculty of Biology, University of Science, Vietnam National University, Ho Chi Minh City, Vietnam; 2 Department of Animal Biotechnology, Institute of Tropical Biology, Vietnam Academy of Science and Technology, Ho Chi Minh City, Vietnam; 3 School of Biotechnology, International University, Vietnam National University, Ho Chi Minh City, Vietnam; 4 Department of Immunology, Vietnam Military Medical University, Ha Noi City, Vietnam

**Keywords:** Apoptosis, Doxorubicin, Hepatocellular Carcinoma Cells, Small Interfering RNA, *Vascular Endothelial Growth Factor*

## Abstract

**Objective:**

Hepatocellular carcinoma (HCC), one of the most common cancers worldwide, is resistant to anticancer drugs. Angiogenesis is a major cause of tumor resistance to chemotherapy, and vascular endothelial growth factor (VEGF) is a key regulator of angiogenesis. The purpose of this study is to investigate the impact of small-interfering RNA targeting *VEGF* gene (VEGF-siRNA) on chemosensitivity of HCC cells *in vitro*.

**Materials and Methods:**

In this experimental study, transfection was performed on Hep3B cells. After transfection with siRNAs, *VEGF* mRNA and protein levels were examined. Cell proliferation, apoptosis and anti-apoptotic gene expression were also analyzed after treatment with VEGF-siRNA in combination with doxorubicin in Hep3B cells.

**Results:**

Transfection of VEGF-siRNA into Hep3B cells significantly reduced the expression of *VEGF* at both mRNA and protein levels. Combination therapy with VEGF-siRNA and doxorubicin more effectively suppressed cell proliferation and induced apoptosis than the respective monotherapies. This could be explained by the significant downregulation of *B-cell lymphoma 2 (BCL-2)* and *SURVIVIN*.

**Conclusion:**

VEGF-siRNA enhanced the chemosensitivity of doxorubicin in Hep3B cells at least in part by suppressing the expression of anti-apoptotic genes. Therefore, the downregulation of *VEGF* by siRNA combined with doxorubicin treatment has been shown to yield promising results for eradicating HCC cells.

## Introduction

Primary hepatocellular carcinoma (HCC) is the fifth most common malignancy worldwide and the second cause of cancer-related deaths. The major therapeutic strategies in solid tumors as well as HCC are excision of the primary tumor, followed by radiotherapy or chemotherapy (1). To date, there are few effective chemotherapeutic agents for this highly malignant cancer. Among the approved anticancer drugs, doxorubicin is perhaps the most widely used traditional chemotherapeutic drug to treat HCC (2). The use of doxorubicin to combat HCC is encouraging, but unfortunately, subsequent studies have been disappointing with low response rates (3, 4) and significant side effects (5). In addition, a recent study has demonstrated that HCC has high immortality partly because of the development of drug resistance during chemotherapy treatment (6). Thus, understanding molecular mechanisms of drug resistance for enhancing the therapeutic efficacy of anti-tumor drugs in HCC treatment are required.

HCC is a highly vascularized tumor that expresses extensive amounts of vascular endothelial growth factor (VEGF) to form numerous blood vessels in order to receive an adequate blood supply for tumor growth. Consequently, HCC progression is correlated with tumor angiogenesis (7). Angiogenesis is governed differentially by multiple factors that include growth factors, cytokines, chemokines, enzymes, and adhesion molecules - the most important of which is *VEGF* (8). Notably, previous studies have indicated that the overexpression of *VEGF* in tumor cells contributes to drug resistance, indicating an association between *VEGF* expression and drug resistance in cancer cells (9-11). Several studies have reported that *VEGF* is frequently expressed in HCC (12, 13). In addition, *VEGF* protein was identified as a key hypoxia- induced angiogenic stimulator in liver cancer (14). Bevacizumab, a humanized monoclonal antibody against *VEGF* protein, has been used in the treatment of advanced HCC, either as a single agent (15) or in combination with chemotherapeutic agents (16, 17). However, the use of anti-VEGF antibodies is responsible for unexpected toxic side effects, especially in terms of thromboembolic events and bleeding that require further investigation (15). It is therefore a challenge to explore a new approach that inhibits *VEGF* expression to identify novel drug targets.

Recently, following the rapid advances in molecular biology, many new therapeutic strategies for treating liver cancer at the genetic level have been developed. In particular, RNA interference (RNAi) may represent a promising therapeutic strategy (14, 18). RNAi is a natural sequence specific post-transcriptional gene regulatory mechanism in which activation of an intracellular pathway triggered by small-interfering RNA (siRNA) of 21–23 nucleotides (nt), leads to gene silencing through degradation of a homologous target mRNA (19). Another unique advantage of RNAi is that non-druggable protein targets can also be efficiently knocked-down and possibly achieve therapeutic effects (20). Therefore, RNAi-based therapeutic strategy presents an effective, simple approach to silence a variety of cancer-associated genes. To date, the RNAi targeting *VEGF*-based therapeutics in combination with chemotherapeutic agents have been found to enhance the therapeutic efficacy of an anti-tumor drug to eradicate colorectal cancer cells, bladder cancer cells, breast cancer cells and myeloma cells (21-24). However, the exact role of *VEGF* gene in this process and the underlying molecular mechanisms remain to be fully elucidated.

In this study, small-interfering RNA targeting *VEGF* gene (referred here as VEGF-siRNA) was transferred into hepatocellular carcinoma Hep3B cells to explore its anti-tumor activity. The effects of VEGF-siRNA combined with doxorubicin treatment on cell proliferation, apoptosis and the anti-apoptotic factors were tested. The possible molecular mechanisms were investigated.

## Materials and Methods

This experimental study was done using an HCC cell line, Hep3B (HB-8064), provided from the American Type Culture Collection (ATCC, Rockville, MD, USA) based on the Ethical Committee approval by the Committee for Ethics in Research, University of Science, Vietnam National University.

### Cell culture

Hep3B cells were thawed and cultured in Dulbecco’s Modified Eagle’s Medium-F12 (DMEMF12) supplemented with 10% fetal bovine serum (FBS), 2 mM L-glutamine and 0.5% antibioticmycotic (all purchased from Sigma-Aldrich, St. Louis, MO, USA). The cells were maintained in a humidified atmosphere of 5% CO_2_ at 37˚C.

### Transfection of small-interfering RNA (siRNA)

The sequences of the siRNA targeting *VEGF* (VEGF-siRNA) and mismatched siRNA (CONTsiRNA) are shown in table 1. All siRNAs were synthesized by Bioneer Co., Ltd (Daejeon, Republic of Korea). Each siRNA was resuspended in nuclease free water (Sigma Sigma-Aldrich, St. Louis, MO, USA) and stored at 4˚C until use. VEGF-siRNA and CONT-siRNA were transiently transfected with a Lipofectamine RNAiMAX Transfection Reagent Kit (Invitrogen Inc., Carlsbad, CA, USA) by the reverse transfection protocol. Briefly, for each well of a 24-well plate (Corning Inc., NY, USA), 3 μl of siRNA (20 μM) was mixed with 1 μl transfection reagent and 100 μl Opti-MEM medium supplied by the kit. Then, the siRNA-transfection reagent complex was incubated with 500 μl of diluted cells (5×10^4^ cells/well) for 24-72 hours at 37˚C and 5% CO_2_. The cells without siRNA transfection were used as the control. The siRNA treated and the untreated cells were harvested during 24-72 hour time intervals for transfection efficiency reverse transcription polymerase chain reaction (RT-PCR) analysis.

### Reverse transcription polymerase chain reaction

Total RNA was extracted using the RNeasy Mini Kit (Qiagen, Valencia, CA, USA). The concentration of RNA was measured by a Biophotometer (Eppendorf, Hamburg, Germany). RT-PCR was performed from total RNA with an Access Quick RT-PCR Kit (Promega, Madison, WI, USA), according to the kit’s procedure manual. The sequences of primers are shown in table 2. PCR products were analyzed by electrophoresis with 2% agarose gel (Sigma-Aldrich, St. Louis, MO, USA), visualized with ethidium bromide (EtBr) staining (Sigma-Aldrich, St. Louis, MO, USA) and photographed by a Bioimaging system (GelDoc-It, UVP, Upland, CA, USA).

**Table 1:** Sequences of VEGF-siRNA and CONT-siRNA


siRNA name	Sequences (5ˊ-3ˊ)

VEGF-siRNA	Sense: GCACAUAGGAGAGAUGAGCUUdTdT
	Antisense: AAGCUCAUCUCUCCUAUGUGCUGdTdT
CONT-siRNA	Sense: GCGGAGAGGCUUAGGUGUAdTdT
	Antisense: UACACCUAAGCCUCUCCGCdTdT


VEGF-siRNA; Vascular endothelial growth factor targeted small-interfering RNA and CONT-siRNA; Mismatched small-interfering RNA.

**Table 2:** Sequences of primers for reverse transcription polymerase chain reaction and real-time quantitative-PCR (qRT-PCR)


Primer	Sequences (5ˊ-3ˊ)	Product size (bp)

VEGF	F: CCATGAACTTTCTGCTGTCTT	250
	R: ATCGCATCAGGGGCACACAG	
BCL-2	F: CGGTGCCACCTGTGGTCCAC	174
	R: TCCCCCAGTTCACCCCGTCC	
SURVIVIN	F: GGACCGCCTAAGAGGGCGTGC	145
	R: AATGTAGAGATGCGGTGGTCCTT	
β-actin	F : ACACTGTGCCCATCTAGGAGG	680
	R: AGGGGCCGGACTCGTCATACT	


F; Forward, R; Reverse, VEGF; Vascular endothelial growth factor, BCL-2; B-cell lymphoma 2 and β-actin; Beta-actin.

### Real-time quantitative reverse transcription polymerase chain reaction

Real-time qRT-PCR was carried out with a SYBR Green One-Step qRT-PCR Kit (Invitrogen Inc., Carlsbad, CA, USA), according to the kit’s procedure manual. Internal calibration curves were generated by real time software. A melting curve analysis was carried out between 60˚C and 95˚C with a plate read every 0.5˚C after holding the temperature for 20 seconds. The cycle number (Ct) at which the signals crossed a threshold set within the logarithmic phase and the peaks of melting curves were recorded. The relative quantitation of gene expression in terms of fold change was calculated using the 2 ^ΔΔCt^ method (25). Relative expression levels of target genes in each group were calculated by normalizing their Ct value against that of an endogenous reference (*β-actin*) and a calibrator (control cells).

### Western blot

After washing with cold phosphate buffered saline (PBS, Sigma-Aldrich, St. Louis, MO, USA), the cells were lysed by a lysis buffer that contained 0.01 M Tris, pH=7.5, 0.1 M NaCl, 1% Triton X-100, 0.5% sodium deoxycholate, and 0.1% sodium dodecyl sulfate (SDS), with added protease inhibitors (all purchased from Sigma-Aldrich, St. Louis, MO, USA). Total proteins in cell lysates were separated by 10% SDS-polyacrylamide gel electrophoresis (PAGE, Sigma-Aldrich, St. Louis, MO, USA) and transferred to a polyvinylidene fluoride blotting membrane (PVDF, Sigma-Aldrich, St. Louis, MO, USA). The membranes were blocked in blocking bovine serum albumin solution (BSA, Sigma-Aldrich, St. Louis, MO, USA) and incubated with mouse anti-VEGF monoclonal antibody (1:200), mouse anti-BCL-2 monoclonal antibody (1:500) and mouse anti-*SURVIVIN* monoclonal antibody (1:500) (all purchased from Abcam, Cambridge, England, UK) for one hour at room temperature. After washing, the membranes were incubated for 45 minutes with horseradish peroxidase (HRP) -linked goat anti-mouse IgG (1:5000, Sigma-Aldrich, St. Louis, MO, USA). The protein bands were visualized by enhanced chemiluminescence (Sigma-Aldrich, St. Louis, MO, USA). Mouse monoclonal Ab against β-actin (Abcam, Cambridge, England, UK) was used as a housekeeping gene control. Band intensities were semi-quantitatively analyzed by Image J densitometer (NIH, Bethesda, MD, USA).

### Quantitative vascular endothelial growth factor measurement

The amount of *VEGF* in cell supernatants was measured using a human *VEGF* enzyme-linked ammunosorbent assay (ELISA) Kit (Life Technologies, Carlsbad, CA, USA) according to the kit’s procedure manual. The human *VEGF* ELISA kit is a "sandwich" enzyme immunoassay that employs monoclonal and polyclonal antibodies. Quantitation can be determined by constructing an absolute standard curve using known concentrations of human *VEGF* proteins.

### Anti-tumor drug treatment assay

To investigate whether the transfection of VEGFsiRNA increases the chemosensitivity of Hep3B cells, VEGF-siRNA treated cells were plated at a density of 1×10^5^ cells per well in 24-well plates (Corning Inc., NY, USA). After a 24-hour culture period, cells were treated with doxorubicin (Sigma- Aldrich, St. Louis, MO, USA) at 0, 1, 2, and 4 μg/ml for 48 hours. Untreated control was also grown under the same conditions. These cells were used for cell morphology, cell proliferation, apoptosis and anti-apoptotic gene expression analyses.

### Cell morphology

After cells were treated with the indicated concentration of doxorubicin for 48 hours according to the above procedure, cell morphology was photographed by an inverted microscope (Olympus, Tokyo, Japan). In another, the medium was removed; cells were rinsed with PBS and stained using the Hoechst 33258 solution (Sigma-Aldrich, St. Louis, MO, USA) according to the manufacturer’s instructions. Stained nuclei were visualized and photographed using an Olympus fluorescence microscope (Olympus, Tokyo, Japan).

### Cell proliferation assay

Cell proliferation was measured by a Cell Proliferation Reagent WST-1 Assay Kit (Roche, Basel, Switzerland). Briefly, siRNAs transfected cells and control cells were seeded at a concentration of 3×10^3^ cells per well in 96-well plates (Corning Inc., NY, USA). For the indicated time, WST-1 solution was applied at 10 μl per well and incubated for 4 hours at 37˚C and 5% CO_2_. The absorbance [also called optical density (OD)] was measured with a microplate ELISA reader (BioTek, Winooski, VT, USA) at 450 nm. Viability and inhibition rate were calculated according to the following equations, respectively. Viability (%)=(OD treated/OD medium)×100%. Inhibition rate (%)=(1- OD treated/OD control)×100%.

### Clonogenic survival assay

The clonogenic survival assay was used to determine the capacity for cell survival and proliferation after radiation or chemotherapy. After treatment with siRNAs, cells were seeded at a density of 100 cells per well in six-well plates in complete medium followed by treatment with doxorubicin. After 24 hours, the medium was replaced with fresh medium and incubated for an additional 10 days. Clones were fixed with methanol and stained with crystal violet (Sigma-Aldrich, St. Louis, MO, USA) for approximately 15 minutes. Stained clones that had more than 50 cells were counted at low magnification and cloning efficiency calculated as follows: cloning efficiency=(clone number/total cell number)×100%.

### Apoptosis assay

Apoptosis was investigated by flow cytometry using annexin V- fluorescein isothiocyanate (annexin V-FITC) and propidium iodide (PI, BD Biosciences, Franklin Lakes, NJ, USA). Briefly, the cell concentration was initially adjusted to 1×10^6^ cells/ml and then 1 ml of the cell suspension was taken and centrifuged at 500×g for 10 minutes at 4˚C. The pellet was rinsed twice with PBS and then re-suspended in a proper volume of binding buffer so that the cell concentration was 5×10^4^x cells/ml. After addition of 10 μl annexin V-FITC and 5 μl PI (Sigma-Aldrich, St. Louis, MO, USA) followed by gentle mixing, a 15 minute reaction was initiated at room temperature in the dark. Subsequently, 300 μl binding buffer was added and flow cytometry using CellQuest Pro software (BD Biosciences, Franklin Lakes, NJ, USA) was performed to detect the rate of cell apoptosis (%).

### Statistical analysis

Each experiment was performed in triplicate for all data. Data were expressed as mean ± standard error of the mean (SEM). Statistical comparisons were performed using the Student’s t test and ANOVA. P values<0.05 were considered to be statistically significant.

## Results

### Effects of vascular endothelial growth factor-small-interfering RNA on *VEGF* expression in Hep3B cells

**Fig.1:**
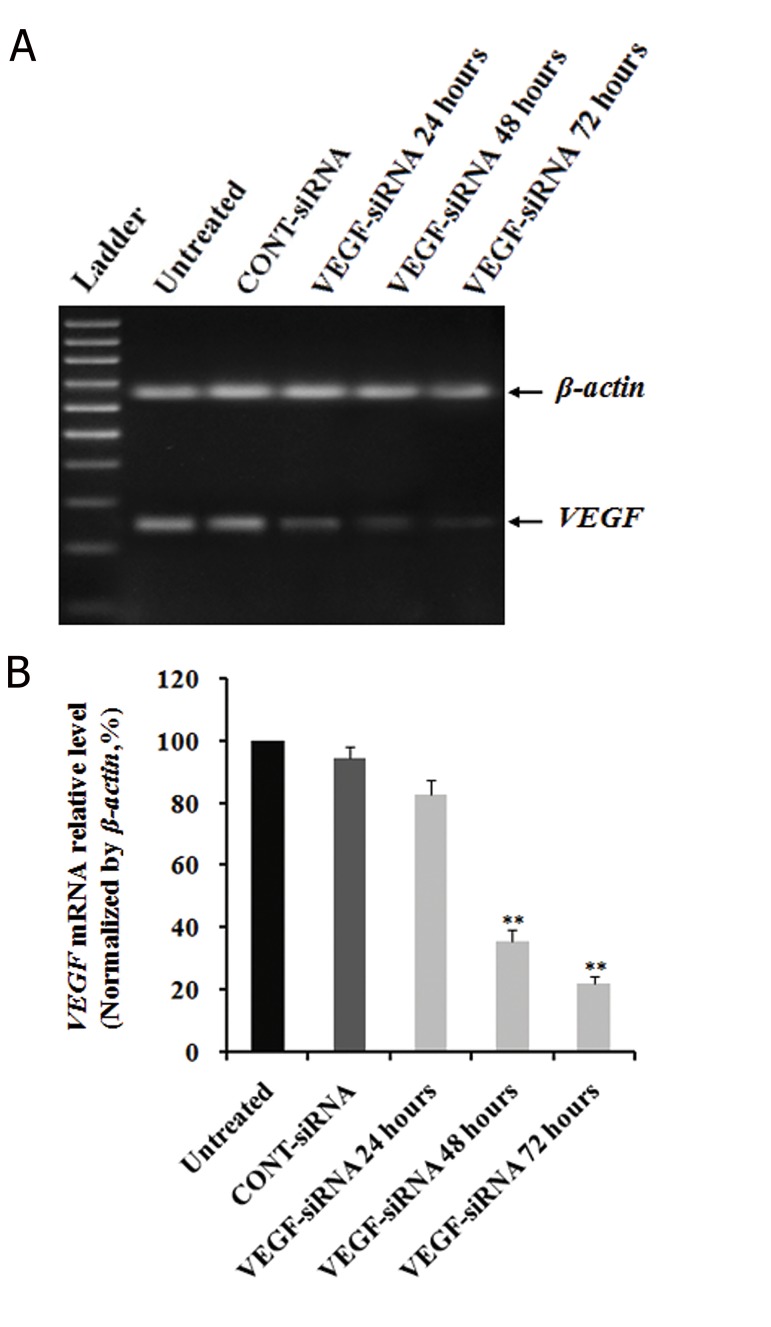
Effects of VEGF-siRNA on *VEGF* mRNA expression in Hep3B cells. Cells were transfected with VEGF-siRNA and CONT-siRNA, then harvested at indicated times. Total RNA was extracted from cells at the indicated time after siRNA transfection. A. Electrophoretic profile of PCR products of the *VEGF* (250 bp) and β-actin (680 bp) genes. β-actin was used as a housekeeping gene control and B. Quantitative analyses of *VEGF* mRNA levels were measured by real-time quantitative PCR (qRT-PCR). mRNA expression of *VEGF* was normalized with β-actin. Each bar represents the mean value ± standard deviation (SD) of triplicate. **; P<0.01 compared to untreated cell group. VEGF-siRNA; Vascular endothelial growth factor targeted smallinterfering RNA, CONT-siRNA; Mismatched small-interfering RNA and PCR; Polymerase chain reaction.

To address if *VEGF* could serve as a therapeutic target for this cancer, we transfected Hep3B cells with VEGF-siRNA and CONT-siRNA. Subsequently, the efficiency of *VEGF* silencing was determined by analysis of *VEGF* mRNA and *VEGF* protein levels. The results of RT-PCR on total RNA obtained from post-transfection cells with siRNAs indicated downregulation of *VEGF* mRNA compared to untreated and CONT-siRNA treated cells. The density of the *VEGF* bands showed that *VEGF* mRNA expression was blocked after 24 hours with stable inhibition up to 72 hours after transfection (Fig.1A). In addition, the results of real-time qRT-PCR analysis indicated that mRNA expression level of *VEGF* in VEGF-siRNA transfected cells began to alter within 24 hours after transfection from 100% to 80.85 ± 4.67%. There was a strong decrease after 48 hours (35.82 ± 3.35%) and 72 hours (21.87 ± 2.69%) compared to untreated cells (P<0.01). We did not observe much alteration in CONT-siRNA transfected cells during 72 hours after transfection (94.68 ± 4.14%, Fig.1B). These values indicated that VEGF-siRNA triggered a 79.13 ± 2.69% decrease in *VEGF* mRNA expression in compared to untreated cells (P<0.01), whereas CONT-siRNA downregulation was approximately 5.32 ± 4.14% at 72 hours.

**Fig.2:**
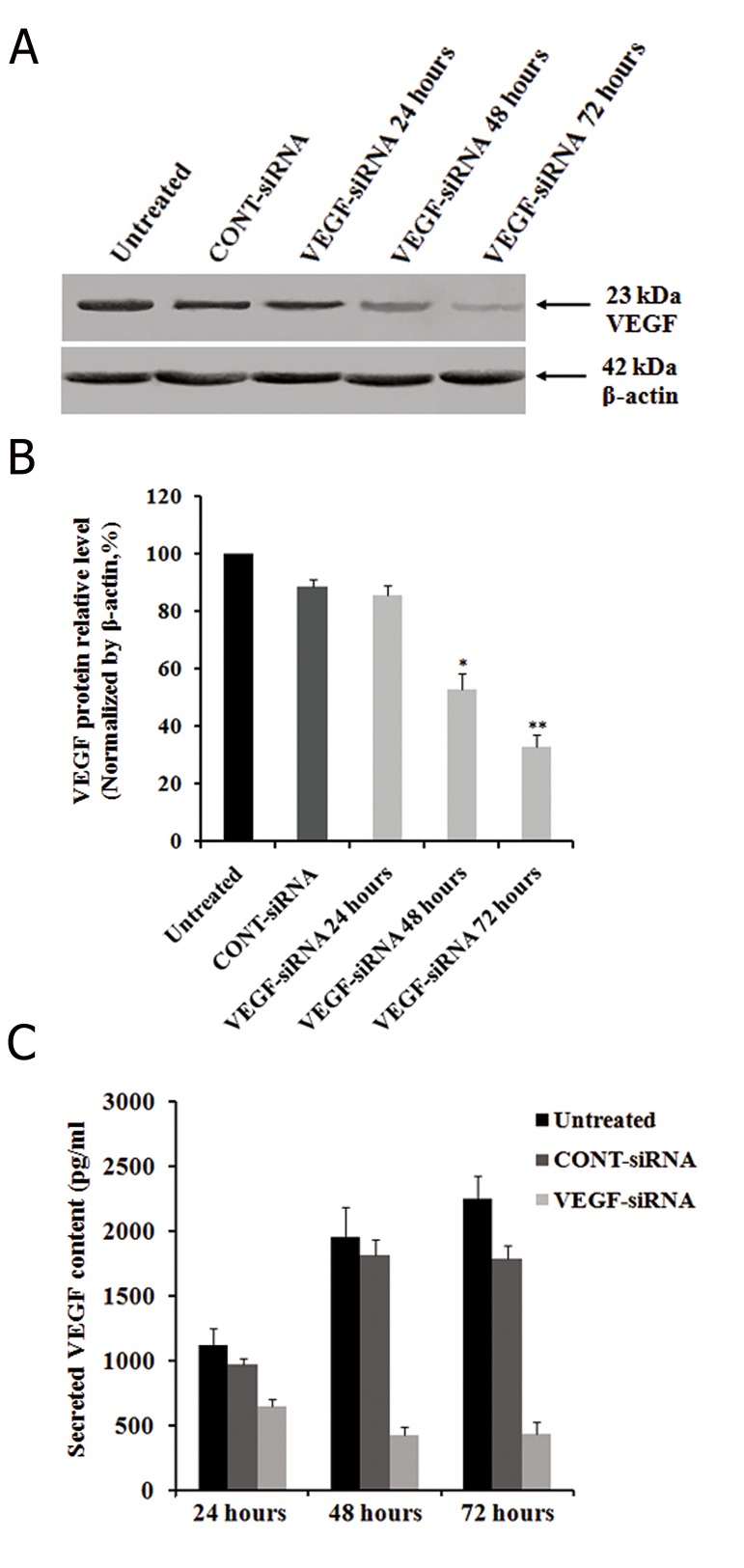
Effects of VEGF-siRNA on VEGF production and secretion in Hep3B cells. Total cellular protein was extracted from cells at an indicated time after siRNA transfection. After electrophoresis and electrotransfer, the membranes were incubated with mouse Ab against VEGF followed by HRP-linked goat anti-mouse IgG. A. Western blot analysis of VEGF protein expression in Hep3B cells. β-actin was used as a housekeeping gene control. The size of each protein was indicated. B. VEGF downregulated Hep3B cells exhibited decreased expression of VEGF protein as confirmed by densitometric analysis and C. The cell culture supernatants were collected at different time intervals and the secreted VEGF concentrations measured by a quantitative VEGF ELISA kit. Each bar represents the mean value ± standard deviation (SD) of triplicate analyses. *; P<0.05, **; P<0.01 compared to untreated cell group, VEGF-siRNA; Vascular endothelial growth factor targeted small-interfering RNA and ELIZA; Enzyme-linked ammunosorbent assay.

The regulatory effects of VEGF-siRNA on *VEGF* protein expression were determined by Western blot at different time intervals after transfection. The results showed that VEGF-siRNA transfected cells expressed significantly less *VEGF* protein than untreated cells or CONT-siRNA treated ones (Fig.2A). Densitometric analyses also confirmed that *VEGF* expression in post-transfected cells was effectively inhibited by VEGF-siRNA at protein levels by 14.35 ± 3.42% after 24 hours; the inhibition was stable up to 72 hours at the protein level (67.25 ± 4.25%), compared to untreated cells (P<0.01), while it was downregulated by CONT-siRNA at protein levels by 11.66 ± 2.87% at 72 hours (Fig.2B). The inhibitory effect of VEGF-siRNA was shown to be specific because siRNA oligos did not cause a nonspecific downregulation of gene expression as demonstrated by the *β-actin* control. Downregulation of *VEGF* protein was confirmed by ELISA. Results indicated that the amount of secreted *VEGF* in untreated cells was approximately 1120 ± 127 pg/ml at 24 hours, 1960 ± 225 pg/ml at 48 hours and 2250 ± 175 pg/ml at 72 hours. However, specific VEGF-siRNA remarkably inhibited *VEGF* production and secretion to 645 ± 55 pg/ml at 24 hours, 425 ± 65 pg/ml at 48 hours and 435 ± 95 pg/ml at 72 hours. CONTsiRNA did not show significant effects on *VEGF* secretion compared to untreated cells as follows: 970 ± 45 pg/ml at 24 hours, 1815 ± 125 pg/ml at 48 hours and 1785 ± 105 pg/ml at 72 hours (Fig.2C).

### Effects of vascular endothelial growth factor-small-interfering RNA in combination with doxorubicin on morphological changes in Hep3B cells

**Fig.3:**
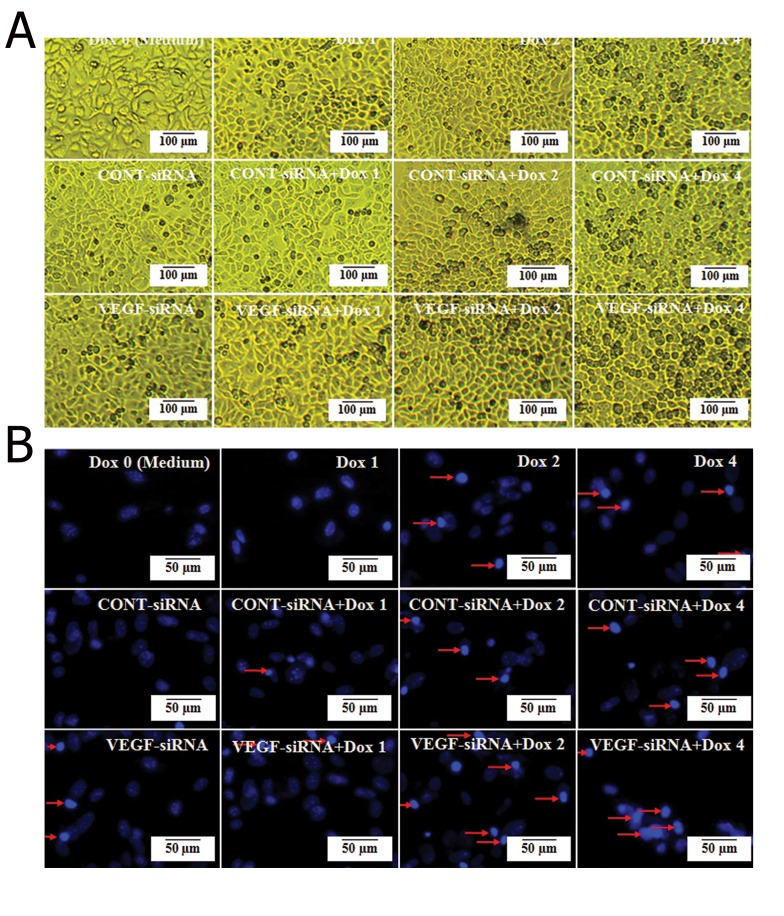
Effects of VEGF-siRNA and/or doxorubicin treatment on cell morphology in Hep3B cells. A. Morphological changes of Hep3B cells treated with the indicated concentration of doxorubicin and VEGF-siRNA/CONT-siRNA alone or together, compared to untreated cells (or medium). Observation by the inverted microscope were taken after 48 hours and B. Hep3B cells were treated with the indicated concentration of doxorubicin and VEGF-siRNA/CONT-siRNA alone or together for 48 hours, then stained with Hoechst 33258. Location of condensed and fragmented nuclei in cells (arrow heads). In this figure, Dox 0; 0 μg/ml (or medium) doxorubicin, Dox 1; 1 μg/ml doxorubicin, Dox 2; 2 μg/ml doxorubicin, Dox 4; 4 μg/ml doxorubicin, VEGF-siRNA; Vascular endothelial growth factor targeted small-interfering RNA and CONT-siRNA; Mismatched small-interfering RNA.

We used an inverted microscope to determine whether the combination of VEGF-siRNA/CONT-siRNA and doxorubicin could affect Hep3B cellular morphology. The observations revealed that exposure of Hep3B cells in VEGF-siRNA combined with doxorubicin for 48 hours displayed significant morphology alterations. We observed no obvious changes in cells treated with 0 μg/ml doxorubicin (medium), 1 μg/ml doxorubicin and VEGF-siRNA/CONT-siRNA alone treated groups. In the 2 μg/ml and 4 μg/ml doxorubicin treated groups, the cells began to shrink and floating cells appeared in the culture medium. In the VEGF-siRNA combined with 2 μg/ml or 4 μg/ml doxorubicin treated groups, most Hep3B cells lost contact with the surrounding cells and additional floating cells emerged. Cell survival decreased significantly compared to the untreated cell group. In the CONT-siRNA combined with doxorubicin treated groups, there were no significant phenotypic differences compared to doxorubicin alone treated cell groups (Fig.3A).

Nuclei stained with Hoechst 33258 showed nuclear chromatin condensation in the VEGF-siRNA and doxorubicin alone or combined treated cells, which was typical of apoptotic cells. VEGF-siRNA combined with 2 μg/ml or 4 μg/ml doxorubicin treated cells showed a more obvious change, while untreated cells had diffuse uniform fluorescence (Fig.3B). The results demonstrated that VEGF-siRNA in combination with doxorubicin affected cell morphology of Hep3B cells and accelerated cell death.

### Effects of vascular endothelial growth factor-small-interfering RNA in combination with doxorubicin on proliferation in Hep3B cells

**Fig.4:**
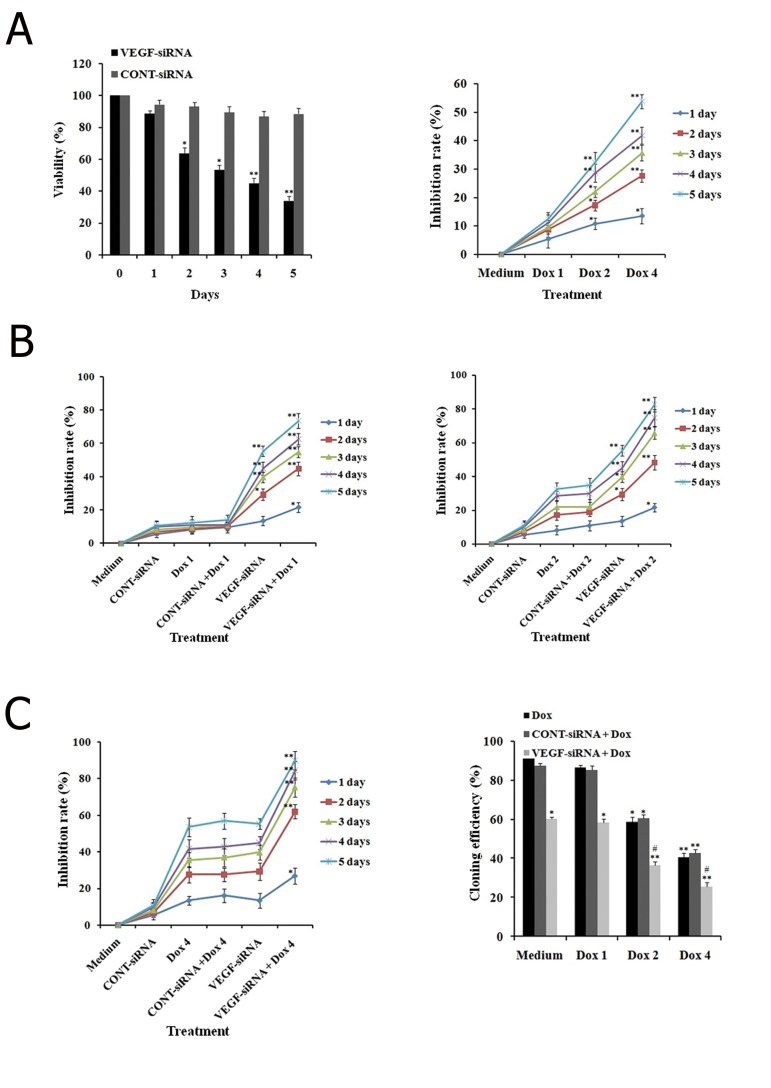
Effects of VEGF-siRNA and/or doxorubicin treatment on growth and colony formation in Hep3B cells. **A**. Cell viability of Hep3B cells treated with VEGF-siRNA or CONT-siRNA for five days in WST-1 assay. Cell viability is expressed as the percentage of control cells (or medium), **B**. Hep3B cells treated with the indicated concentration of doxorubicin at a specified time in the WST-1 assay. Results are presented as the inhibitory ratio of doxorubicin treated cells, **C, D, E**. Hep3B cells treated with VEGF-siRNA/CONT-siRNA in combination with the indicated concentration of doxorubicin for specified time. Inhibition was determined by the WST-1 assay and F. Effects of VEGF-siRNA and/or doxorubicin treatment on the inhibition of cell proliferation were confirmed by the cloning efficiency. Each bar represents the mean value ± standard deviation (SD) of triplicate experiments. *; P<0.05, **; P<0.01 compared to the control cell group (medium) for A, B and F or doxorubicin alone treated cell group for C, D, E, #; P<0.05 compared to the VEGF-siRNA treated cell group, Dox 0; 0 μg/ml (or medium) doxorubicin, Dox 1; 1 μg/ml doxorubicin, Dox 2; 2 μg/ml doxorubicin, Dox 4; 4 μg/ml doxorubicin, VEGF-siRNA; Vascular endothelial growth factor targeted small-interfering RNA and CONT-siRNA; Mismatched small-interfering RNA.

In order to evaluate the effects of VEGF-siRNA on viability of Hep3B cells, we treated the cells for an indicated period of time. VEGF-siRNA significantly decreased the viability of Hep3B cells in a time-dependent manner. These findings indicated that Hep3B cells were sensitive to VEGF-siRNA. We also compared the cytotoxicity of CONT-siRNA and VEGF-siRNA toward Hep3B cells. VEGF-siRNA, but not CONT-siRNA, directly mediated cytotoxicity toward Hep3B cells (Fig.4A).

We evaluated the inhibitory effect of doxorubicin on Hep3B cells. Proliferation of cells following treatment with designated concentrations of doxorubicin was detected by the WST-1 and clonogenic survival assays for a specified period of time. It was clear that doxorubicin alone significantly reduced Hep3B cell growth and the inhibitory effect was dose and time-dependent (Fig.4B). However, the effects of doxorubicin on Hep3B cells were not observable at the concentration of 1 μg/ml. At 4 μg/ml, inhibition by doxorubicin on cell proliferation became apparent, the inhibition rate increased from 13.51 ± 2.67% at day 1 to 53.82 ± 2.46% at day 5. In addition, cloning efficiency declined significantly in cells after treatment with 4 μg/ml doxorubicin compared to untreated cells (P<0.01).

In order to evaluate the synergistic effect of VEGF-siRNA and doxorubicin on Hep3B cells, cells treated with VEGF-siRNA/CONT-siRNA in the presence or absence of doxorubicin were assayed by WST-1 and clonogenic survival. The effects of doxorubicin were noticeable in *VEGF* downregulated cells. As illustrated in figure 4C, after 5 days of treatment, VEGF-siRNA combined with 1 μg/ml doxorubicin increased the inhibition rate (73.55 ± 4.52%) compared to VEGF-siRNA alone (55.37 ± 3.04%, P<0.01) or doxorubicin alone (12.40 ± 4.02%, P<0.01). However, there was no significant difference in inhibition of cell growth between CONT-siRNA plus 1 μg/ml doxorubicin or CONT-siRNA and doxorubicin alone. To further determine if VEGF-siRNA could enhance the chemosensitivity of doxorubicin-treated Hep3B cells, VEGF-siRNA/CONT-siRNA treated cells as well as untreated cells were treated with higher doses of doxorubicin (2 and 4 μg/ml). The inhibition rate for VEGF-siRNA plus 2 μg/ml doxorubicin was 82.78 ± 4.32%, whereas it was 90.08 ± 4.07% in the 4 μg/ml doxorubicin group. For CONT-siRNA plus 2 μg/ml doxorubicin, the inhibition rate was 34.71 ± 3.43%, in the 4 μg/ml doxorubicin group, the inhibition rate was 57.02 ± 4.14%. In the 2 μg/ml doxorubicin alone group, the inhibition rate was 32.23 ± 4.21% and the 4 μg/ml doxorubicin alone group showed an inhibition rate 56.72 ± 3.28% (Fig.4D, E). In addition, the *VEGF* downregulated cells showed no signs of proliferation, with necrosis observed at day 3 after doxorubicin treatment (data not shown). Treatment with a series of doses of doxorubicin in the presence of VEGF-siRNA increased inhibition compared to treatment with doxorubicin and/or CONT-siRNA, which further supported the synergistic effect. VEGF-siRNA transfer could increase doxorubicin chemosensitivity of Hep3B cells. Of note, the synergistic cytotoxic effect was effective, even at a low dose (1 μg/ml) compared to the control. These results were also supported by the clonogenic survival assay. A highly-significant decline in cloning efficiency was observed in VEGFsiRNA plus doxorubicin treated cell groups compared to VEGF-siRNA alone or doxorubicin alone treated groups after similar treatment conditions (Fig. 4F).

### Effects of vascular endothelial growth factor-smallinterfering RNA in combination with doxorubicin on apoptosis in Hep3B cells

**Fig.5:**
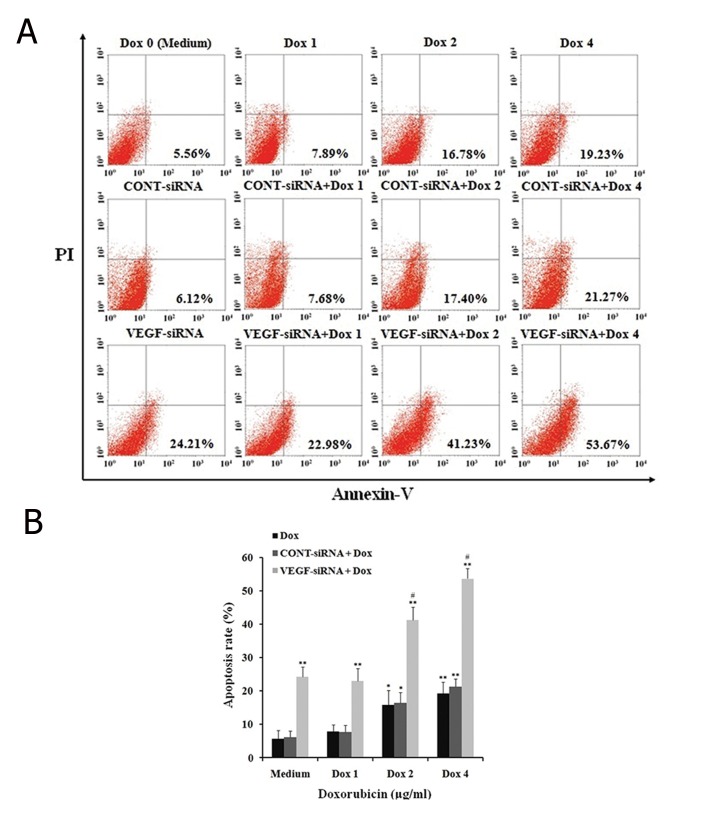
Effect of VEGF-siRNA and/or doxorubicin treatment on apoptosis in Hep3B cells. A. Cell apoptosis was detected using annexin V-FITC/ propidium iodide (PI) staining and flow cytometry analysis. Fluorescence intensity of annexin V/FITC is plotted on the x-axis, and PI is plotted on the y-axis. Cells in the lower left (LL) quadrant represent survivals, lower right (LR) quadrant represent early apoptosis, the upper right (UR) quadrant represent necrosis or post-apoptotic and the upper left (UL) quadrant represent detection of error allowed and B. The percentage of apoptotic cells examined by annexin V-FITC/PI staining and flow cytometry analysis. Each bar represents the mean value ± standard deviation (SD) intensity of fluorescent positive cells during early apoptotic events of triplicate experiments. *; P<0.05, **; P<0.01 compared to control cell group (or medium), #; P<0.05 compared to VEGF-siRNA treated cell group, Dox 0; 0 μg/ml (or medium) doxorubicin, Dox 1; 1 μg/ml doxorubicin, Dox 2; 2 μg/ml doxorubicin, Dox 4; 4 μg/ml doxorubicin, VEGF-siRNA; Vascular endothelial growth factor targeted small-interfering RNA and FITC; Fluorescein isothiocyanate.

To evaluate whether VEGF-siRNA enhanced the chemosensitivity of doxorubicin treated Hep3B cells by promoting apoptosis, we analyzed the apoptotic rate of cells following treatment with doxorubicin for 48 hours by annexin V-FITC/PI staining method. As shown in figure 5, VEGF-siRNA combined with doxorubicin promoted apoptosis compared to VEGF-siRNA and doxorubicin alone (Fig.5A). The apoptosis rate of control cells increased gradually with doxorubicin concentrations of 0 μg/ml (5.56 ± 1.62%), 1 μg/ml (7.89 ± 1.97%), 2 μg/ml (16.28 ± 4.23%) and 4 μg/ml (21.23 ± 4.78%). The apoptotic effect was obvious in *VEGF* downregulated cells which showed an increase to 22.98 ± 3.78% when treated with 1 μg/ml doxorubicin. The level of apoptosis rose steadily to 53.67 ± 3.08% at a concentration of 4 μg/ml doxorubicin. However, this result was not reproduced by CONT-siRNA, which showed no significant difference in apoptosis rate between CONT-siRNA in combination with the doxorubicin treated groups and doxorubicin alone groups (Fig.5B). The finding suggested that VEGF-siRNA increased chemosensitivity of Hep3B cells by inducing apoptosis.

### Effects of vascular endothelial growth factor-small-interfering RNA in combination with doxorubicin on anti-apoptotic gene expression in Hep3B cells

**Fig.6:**
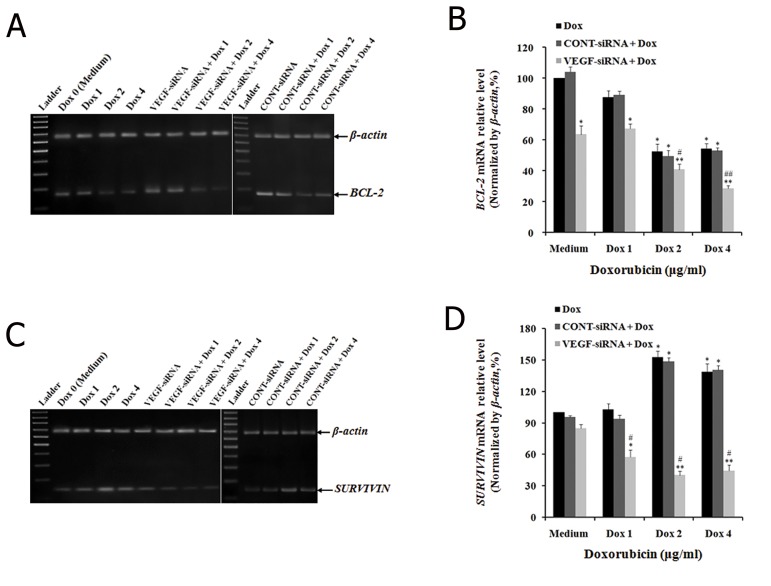
Effect of VEGF-siRNA and/or doxorubicin treatment on BCL-2 and SURVIVIN mRNA expression in Hep3B cells. A, C. mRNA expressions of BCL-2 and SURVIVIN in cells detected by reverse transcription polymerase chain reaction (RT-PCR) after treatment with the indicated concentration of doxorubicin for 48 hours. Electrophoretic profile of PCR products of BCL-2 (174 bp), SURVIVIN (145 bp) and β-actin (680 bp) genes. β-actin was used as a housekeeping gene control and B, D. mRNA levels of BCL-2 and *SURVIVIN* in cells determined by real-time quantitative PCR (qRT-PCR). mRNA expressions of these genes were normalized with β-actin. Each bar represents the mean value ± standard deviation (SD) of triplicate experiments. *; P<0.05, **; P<0.01 compared to control cell group (or medium), #; P<0.05, ##; P<0.01 compared to VEGF-siRNA treated cell group, Dox 0; 0 μg/ml (or medium) doxorubicin, Dox 1; 1 μg/ml doxorubicin, Dox 2; 2 μg/ml doxorubicin, Dox 4; 4 μg/ml doxorubicin and VEGF-siRNA; Vascular endothelial growth factor targeted small-interfering RNA

**Fig.7:**
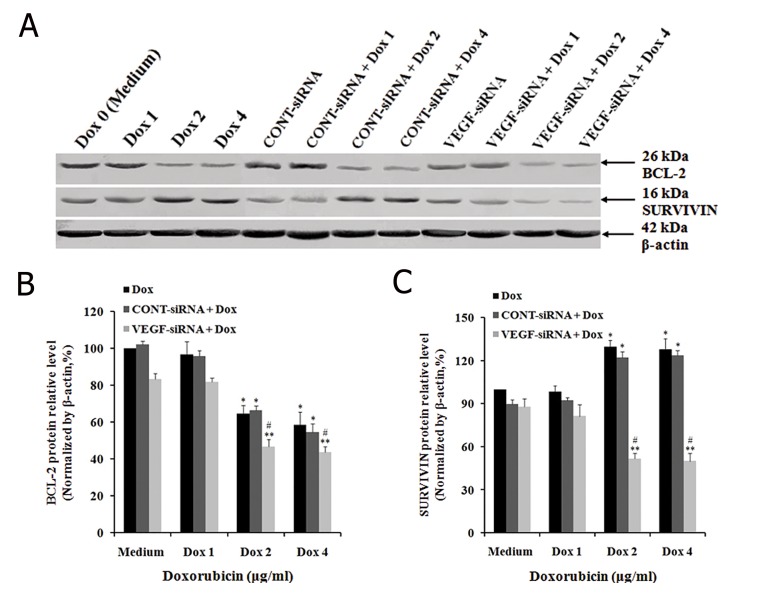
Effect of VEGF-siRNA and/or doxorubicin treatment on BCL-2 and *SURVIVIN* protein expression in Hep3B cells. A. Protein expressions of BCL-2 and *SURVIVIN* in Hep3B cells measured by Western blot analyses after treatment with the indicated concentration of doxorubicin for 48 hours. β-actin was used as a housekeeping gene control. The size of each protein was indicated and B, C. Densitometric analysis of these three proteins relative to β-actin. Each bar represents the mean value ± standard deviation (SD) of triplicate experiments. *; P<0.05, **; P<0.01 compared to control cell group (or medium), #; P<0.05 compared to VEGF-siRNA treated cell group, Dox 0; 0 μg/ml (or medium) doxorubicin, Dox 1; 1 μg/ml doxorubicin, Dox 2; 2 μg/ml doxorubicin, Dox 4: 4 μg/ml doxorubicin and VEGF-siRNA; Vascular endothelial growth factor targeted small-interfering RNA.

We sought to determine whether VEGF-siRNA in combination with doxorubicin promoted the chemosensitivity of doxorubicin in Hep3B cells by regulating the expression of BCL-2 by incubating control and VEGF-siRNA/CONT-siRNA treated cells with an indicated concentration of doxorubicin. After 48 hours, the expression of BCL-2 was determined at both mRNA and protein levels. The results showed that VEGF-siRNA and doxorubicin alone could decrease expression of BCL-2 at both mRNA and protein levels within Hep3B cells (Figes.6, 7). The band intensity of RT-PCR products showed decreased expression levels of BCL-2 mRNA in cells treated with 2 μg/ml and 4 μg/ml of doxorubicin, VEGF-siRNA alone, or the combination of doxorubicin and VEGF-siRNA compared to untreated cells (Fig.6A). The mRNA levels of BCL-2 were downregulated as follow in control cells treated with various concentrations of doxorubicin: 12.44 ± 4.37% (1 μg/ml), 47.54 ± 5.02% (2 μg/ml) and 45.68 ± 3.23% (4 μg/ml) in control cells. BCL-2 mRNA level downregulated by 36.48 ± 5.52% in VEGF-siRNA alone treated cells compared to untreated cells (P<0.05, Fig.6C). Similarly, BCL-2 protein expression was measured by Western blot (Fig.7A). The protein levels of BCL-2 were downregulated by 3.44 ± 4.98% (1 μg/ml), 35.42 ± 4.43% (2 μg/ml), 41.47 ± 7.02% (4 μg/ml) and 16.58 ± 2.87% (VEGF-siRNA alone) compared to untreated cells (P<0.05, Fig.7B). In addition, the combination of VEGF-siRNA and doxorubicin was more effective in suppressing BCL-2 than respective treatments. The mRNA levels of BCL-2 were downregulated by 32.58 ± 3.02%, 59.24 ± 3.58% and 71.54 ± 1.88% in cells treated with a combination of VEGF-siRNA and doxorubicin (1 μg/ml, 2 μg/ml, 4 μg/ml) compared to untreated cells (P<0.01, Fig.6C). The protein levels of BCL-2 were downregulated by 18.23 ± 2.14%, 53.22 ± 3.98% and 56.44 ± 7.02% in cells treated with a combination of VEGF-siRNA and doxorubicin (1 μg/ml, 2 μg/ml, 4 μg/ml) compared to untreated cells (P<0.01, Fig.7B). However, the combination of CONT-siRNA and doxorubicin had the same influences on *BCL-2* expression at both the mRNA and protein levels when compared to doxorubicin alone. These results showed that VEGF-siRNA alone or doxorubicin alone, and VEGF-siRNA in combination with doxorubicin could induce apoptosis of Hep3B cells by downregulating expression of *BCL-2*.

We sought to examine whether VEGF-siRNA in combination with doxorubicin promoted the chemosensitivity of doxorubicin in Hep3B cells by regulating the expression of *SURVIVIN*. We performed RT-PCR, real-time qRT-PCR and Western blot analyses with the same method. The band intensity of RT-PCR products showed that mRNA expression of *SURVIVIN*. decreased in cells treated with VEGF-siRNA alone or VEGF-siRNA in combination with doxorubicin. However, mRNA expression of *SURVIVIN*. increased in cells treated with doxorubicin alone (Fig.6B). These results were also confirmed by real-time qRT-PCR and Western blot analyses. For the VEGF-siRNA alone treated cell group, the relative mRNA expression level of *SURVIVIN*. was 87.74 ± 3.67%, lower than untreated cells (100%). The results showed that VEGF-siRNA alone could downregulate the expression of *SURVIVIN*. For the doxorubicin treated cell groups, the relative mRNA expression levels of *SURVIVIN*. were 102.57 ± 5.78% (1 μg/ml), 152.67 ± 6.06% (2 μg/ml) and 138.86 ± 7.76% (4 μg/ml), which was higher than untreated cells. The results showed that doxorubicin alone could upregulate the expression of *SURVIVIN*. VEGF-siRNA combine with doxorubicin had the following relative mRNA expression levels of *SURVIVIN*: 57.42 ± 7.03% (1 μg/ml), 40.23 ± 4.07% (2 μg/ml) and 44.543 ± 5.58% (4 μg/ml) (Fig.6D). Measurement of expression of *SURVIVIN*. protein by Western blot analysis after treatment indicated similar results (Fig.7A, C). However, there was no significant difference in *SURVIVIN*. expression between CONT-siRNA in combination with doxorubicin treated cells and doxorubicin alone treated cells. These clear results indicated that VEGF-siRNA in combination with doxorubicin could promote the chemosensitivity to doxorubicin in Hep3B cells by downregulating the expression of *SURVIVIN*. when compared with doxorubicin alone.

## Discussion

As one of the most important angiogenesis-stimulating factors, *VEGF* contributes to cancer progression through its action of tumor neovascularization, tumor invasion, metastasis and drug resistance (8, 10, 26). Therefore, *VEGF* gene is obviously the target of intense research for the development of novel anticancer therapeutics. siRNA- based approaches are efficient tools for the specific inhibition of gene expression (19). In our study, the results have indicated that transfection of VEGF-siRNA into Hep3B cells reduced the expression of *VEGF* at both mRNA and protein levels. Our observations were consistent with previous reports that also used VEGF-siRNA to suppress *VEGF* expression in cancer cells (14, 21, 24, 27, 28).

Drug tolerance is a major obstacle to the effective treatment of cancer. HCC is extremely insensitive to chemotherapy (14, 15). It is known that VEGF-concerned angiogenesis inside solid tumors is a major cause of tumor resistance to chemotherapy. *VEGF* has been shown to mediate cytoprotection against antitumor drug-caused cell death and significantly increase cell survival in tumors resistant to anti-tumor drugs (9, 29, 30). In seeking potential strategies to combat the resistance of HCC to doxorubicin, the present study has shown that VEGF-siRNA exerted its anti-tumor effect by downregulating the expression of *VEGF*, which resulted in significantly increased doxorubicin sensitivity in *VEGF* downregulated cells compared to the original cells. Even at high concentrations, the drug failed to kill normal cancer cells, whereas it killed the VEGF-siRNA transfected cells at lower concentrations. This implied that the reduction of *VEGF* expression mitigated drug-resistant ability of HCC cells. Our findings have provided further evidence that a correlation exists between drug resistance and *VEGF* expression or signal pathways related to this protein. However, molecular mechanisms leading to death of HCC cells by the combination of VEGF-siRNA and doxorubicin are not well understood and require further investigation.

Induction of apoptosis and expression of anti-apoptotic factors were tested to elucidate the molecular mechanisms by which VEGF-siRNA transfection increased the chemosensitivity of Hep3B cells. Apoptosis is thought to be the major reason for cell death. A previous study has suggested that *VEGF* may play an important role in mediating the development of drug resistance by suppression of tumor cell apoptosis. The potential impacts of *VEGF* on delaying apoptosis and prolonging survival of cancer cells may be indirect through the induction of cytokines or inhibition of apoptosis-associated specific genes (11). In this study, our results have shown that apoptosis was responsible for VEGF-siRNA and/or doxorubicin induced cytotoxicity of Hep3B as seen by the Hoechst 33258 and annexin V-FITC staining methods. These results indicated that VEGF-siRNA directly increased the chemosensitivity of doxorubicin in Hep3B cells by increasing apoptosis. Downregulation of *VEGF* also enhanced chemosensitivity by induction of apoptosis in colorectal cancer and myeloma cells (21, 24). Obviously, this finding further supported previous studies that *VEGF* gene was closely associated with apoptosis in cancer cells.

Angiogenic growth factors may modulate anti-tumor drug sensitivity indirectly through the regulation of the activity of some drug-resistance related genes in tumor cells. The expression of anti-apoptotic factors is also a key mechanism involving drug resistance of cancer cells (31). Members of the anti-apoptotic BCL-2 family are related to tumorigenesis and the sensitivity of chemotherapeutic drugs in tumors. Overexpression of the *BCL-2* is frequently detected in many types of human cancers and promotes resistance to chemotherapy (32). Conversely, the downregulation of *BCL-2* expression may enhance apoptotic response to anticancer drugs (33). In this work, our data have also shown that VEGF-siRNA alone and doxorubicin alone inhibited the expression of *BCL-2* within Hep3B cells. VEGF-siRNA in combination with doxorubicin suppressed the expression of *BCL-2* better than the respective treatments. These results suggested that apoptosis induced by VEGF-siRNA and/or doxorubicin was related to downregulation of *BCL-2*, but the exact molecular mechanism should be further studied.

Similarly, the *SURVIVIN* protein, which belongs to the inhibitors of apoptotic proteins (IAPs), has been implicated in both cell division and inhibition of apoptosis. By inhibiting apoptosis and promoting mitosis, *SURVIVIN* may confer cancer cell survival and growth. *SURVIVIN* differs from the other members of IAP family because it has low or no expression in normal tissues. It is highly expressed in tumor tissues. The induction of apoptosis is generally associated with suppression of *SURVIVIN* within tumor cells (34). In addition, more recent reports have demonstrated that chemoresistance in tumor cells is more closely related with overexpression of *SURVIVIN*; inhibition of *SURVIVIN* expression has been shown to improve their sensitivity to chemotherapy (35, 36). In the present study, our data showed that doxorubicin alone upregulated the expression of *SURVIVIN* within Hep3B cells, which indicated resistance to apoptosis induced by doxorubicin. However, Hep3B cells following treatment with VEGF-siRNA in the presence or absence of doxorubicin downregulated the expression of *SURVIVIN*. These results suggested that VEGF-siRNA promoted the chemosensitivity of doxorubicin in Hep3B cells by downregulating the expression of *SURVIVIN* within Hep3B cells. Based on the results of this study, we have proposed a previously unreported molecular mechanism involved in enhancing chemosensitivity by VEGF-siRNA in HCC cells. However, the efficiency of combination with siRNA and doxorubicin in treatment of HCC in vivo remains unknown. Therefore, using mouse xenograft tumor models to confirm the effects of combined treatment should be investigated in susequent experiments.

## Conclusion

Our results demonstrated that downregulation of anti-apoptotic gene expressions, such as *BCL- 2* and *BCL-2*, led to apoptosis by VEGF-siRNA. This was an important way to enhance chemosensitivity in Hep3B cells, which subsequently led to cell death. Downregulation of *VEGF* by siRNA when combined with doxorubicin treatment yielded promising results for eradicating HCC cells *in vitro*.
